# Enhanced CH_4_/N_2_ Separation Efficiency of UiO-66-Br_2_ through Hybridization with Mesoporous Silica

**DOI:** 10.3390/molecules29122750

**Published:** 2024-06-09

**Authors:** Hu Wang, Ziao Zong, Yadong Zhou, Chaochuang Yin, Yizhu Lei, Renshu Wang, Yuheng Deng, Tingting Wu

**Affiliations:** 1 Guizhou Provincial Key Laboratory of Coal Clean Utilization, School of Chemistry and Materials Engineering, Liupanshui Normal University, Liupanshui 553004, China; 2 School of Laboratory Medicine, Youjiang Medical University for Nationalities, Baise 533000, China; 3 Department of Chemistry, Capital Normal University, Beijing 100048, China

**Keywords:** adsorption, CH_4_/N_2_ separation, SBA-15, metal-organic frameworks, adsorbents

## Abstract

Efficient separation of CH_4_ from N_2_ is essential for the purification of methane from nitrogen. In order to address this problem, composite materials consisting of rod-shaped SBA-15-based UiO-66-Br_2_ were synthesized for the purpose of separating a CH_4_/N_2_ mixture. The materials were characterized via PXRD, N_2_ adsorption–desorption, SEM, TEM, FT-IR, and TGA. The adsorption isotherms of CH_4_ and N_2_ under standard pressure conditions for the composites were determined and subsequently compared. The study revealed that the composites were formed through the growth of MOF nanocrystals on the surfaces of the SBA-15 matrix. The enhancements in surface area and adsorption capacity of hybrid materials were attributed to the structural modifications resulting from the interactions between surface silanol groups and metal centers. The selectivity of the composites towards a gas mixture of CH_4_ and N_2_ was assessed utilizing the Langmuir adsorption equation. The results of the analysis revealed that the U6B2S5/SBA-15 sample exhibited the greatest selectivity for CH_4_/N_2_ adsorption compared to the other samples, with an adsorption selectivity parameter (*S*) of 20.06. Additional research is necessary to enhance the enrichment of methane from CH_4_/N_2_ mixtures using SBA-15-based metal-organic framework materials.

## 1. Introduction

Methane (CH_4_) is identified as one of the six primary greenhouse gases responsible for global warming under the Kyoto Protocol. It possesses a global warming potential twenty-one times greater than that of carbon dioxide (CO_2_) [1,2,3,4,5]. In spite of this, methane remains a valuable energy source, utilized in residential, commercial, and industrial applications due to its cleaner and more cost-effective nature compared to traditional fossil fuels [6,7]. Various gas types, including coalbed methane (CBM), low-grade natural gas, landfill gases, and industrial by-product gases, contain methane as their primary component, along with CO_2_, N_2_, H_2_O, and SO_x_. Although established methods can be used to separate CO_2_, H_2_O, and SO_x_ molecules from methane, removing N_2_ is a challenging process due to their similar kinetic diameters (CH_4_: 3.80 Å and N_2_: 3.64 Å) and polarizabilities (CH_4_: 26.0 × 10^−25^ cm^3^ and N_2_: 17.6 × 10^−25^ cm^3^) [8,9,10,11]. It is worth noting that the methane concentration plays a crucial role in determining the appropriate method for CBM extraction, as a very low concentration would constrain the feasibility of this approach [12,13,14]. Currently, ventilation air methane, particularly those with high N_2_ contents, is currently being emitted into the atmosphere without being captured or only partially utilized as low-quality heating sources, leading to a significant greenhouse effect. Hence, the separation of CH_4_/N_2_ to mitigate methane emissions and produce high-quality fuel gas holds significant importance from both economic and environmental standpoints. In order to address the constraints posed by conventional commercial adsorbents in the context of CH_4_/N_2_ separation, the development of a novel adsorbent is imperative [15,16,17].

In recent decades, the separation and purification of CBM have been extensively investigated using various technologies, including cryogenic distillation, solution absorption [18], membrane separation [19], vacuum swing adsorption (VSA) and pressure swing adsorption (PSA) [20]. Cryogenic distillation is a well-established technology widely employed in CBM separation. Nevertheless, it is characterized by high energy consumption and costs, often necessitating high flow rates to maintain economic viability [21,22]. As an alternative, VSA is regarded as the most cost-effective method due to its reduced resource requirements, particularly in terms of energy consumption and its high level of efficiency [23,24]. Adsorbents are crucial in facilitating the effective separation of methane and nitrogen through VSA technology [25]. Metal-organic frameworks (MOFs) have gained considerable attention within the realm of porous adsorbents due to their intricate design capabilities and exceptional porosity [26,27,28,29,30,31].

Moreover, SBA-15, being a representative mesoporous material, has garnered significant interest in adsorption, separation, and catalysis applications [32,33,34,35,36,37]. In light of the background of CBM application, it is necessary to utilize adsorbents with a high performance-to-cost ratio. The intricate nature of the preparation process, limited yield, and elevated cost associated with metal-organic frameworks (MOFs) serve as constraints on their widespread utilization. Therefore, after evaluating various commercial materials such as activated carbon, a carbon molecular sieve, and a zeolite molecular sieve, a rod-shaped SBA-15 with a significant surface area is chosen as the optimal carrier for the MOF structure [38,39,40].

In the present investigation, UiO-66-Br_2_ was chosen as the MOF material due to its possession of microporous cages and its demonstrated high levels of hydrothermal, thermal, and mechanical stability, as well as its good performance in CH_4_/N_2_ separation. A simple solvothermal method was adopted to disperse the MOF crystals on the surfaces of rod-shaped SBA-15 without any intricate pre-functionalization process, to prepare the composite material. Herein, this study investigates the synthesis, characterization, and adsorption capabilities of UiO-66-Br_2_/SBA-15 materials. The results indicate that these adsorbents are a viable option for selectively adsorbing and enriching CH_4_ from CH_4_/N_2_ mixtures.

## 2. Results and Discussion

### 2.1. Powder X-ray Diffraction (PXRD) Patterns

**Figure 1:**
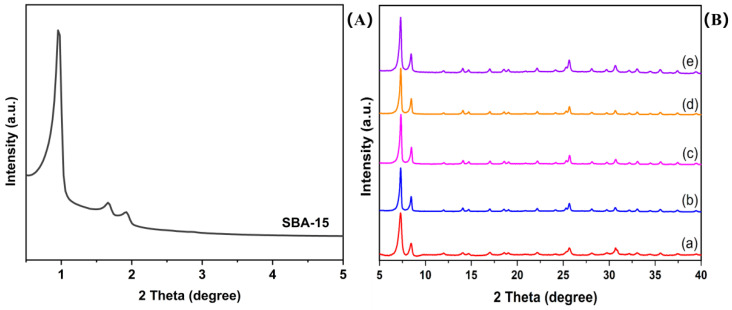
Small-angle X-ray diffraction (SXRD) pattern of SBA-15 (**A**) and wide-angle X-ray diffraction (WXRD) patterns of (a) UiO-66-Br_2_, (b) U6B2S1, (c) U6B2S3, (d) U6B2S5, and (e) U6B2S7 (**B**).

Small-angle X-ray diffraction (SXRD) is a highly effective method for characterizing the structural organization of materials, particularly in the identification of ordered mesoporous structures, making it a prominent technique in material characterization [41,42]. The SXRD patterns of SBA-15 are depicted in Figure 1A, revealing a characteristic pattern featuring a prominent reflection at 2θ approximately 1.0° (corresponding to the (100) plane indicative of a cavity with hexagonal symmetry).

The wide-angle X-ray diffraction (WXRD) patterns of UiO-66-Br_2_ as well as the composites U6B2S1, U6B2S3, U6B2S5, and U6B2S7 are depicted in Figure 1B. The prominent characteristic peaks observed in bare UiO-66-Br_2_ are consistent with those documented in the existing literature [43]. Likewise, the composites exhibited comparable patterns, suggesting that the incorporation of SBA-15 did not impede the development of the UiO-66-Br_2_ component.

### 2.2. Nitrogen Adsorption–Desorption

**Figure 2:**
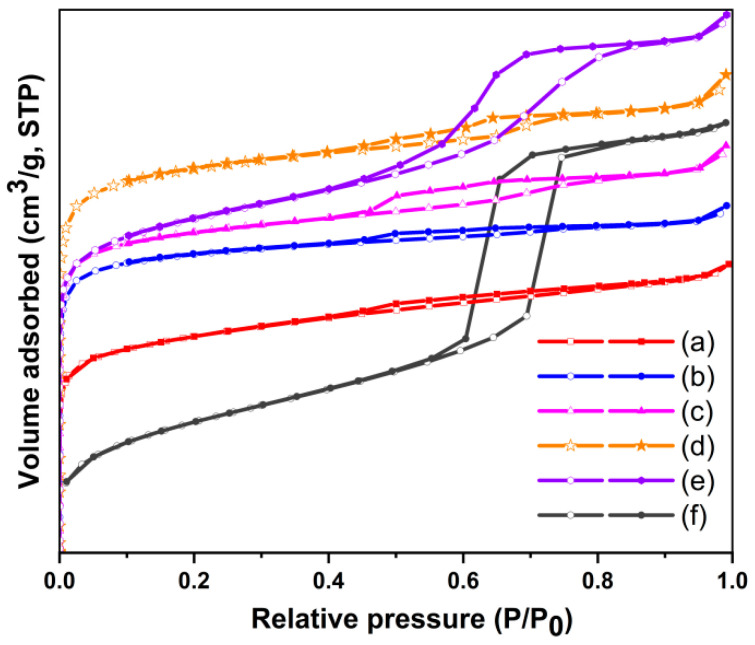
N_2_ adsorption–desorption isotherms of (**a**) UiO-66-Br_2_, (**b**) U6B2S1, (**c**) U6B2S3, (**d**) U6B2S5, (**e**) U6B2S7, and (**f**) SBA-15.

N_2_ adsorption–desorption measurements were conducted on UiO-66-Br_2_, SBA-15, and the UiO-66-Br_2_/SBA-15 composites to assess their textural characteristics, with the resulting isotherms presented in Figure 2. UiO-66-Br_2_ exhibits a type I isotherm, indicative of its microporous nature. The BET surface area of UiO-66-Br_2_ is approximately 515.9 m^2^/g, a value in close agreement with the literature [43,44]. The isotherms of SBA-15 (Figure 2f) exhibited a type IV classification with a H1-type hysteresis loop, indicating the mesoporous nature of the material. The presence of both micropores and mesopores in all hybrid composites was indicated by the observation of type I and type IV isotherms. Furthermore, the hysteresis loops derived from the mesopores exhibited increasing prominence with higher concentrations of SBA-15.

**Figure 3:**
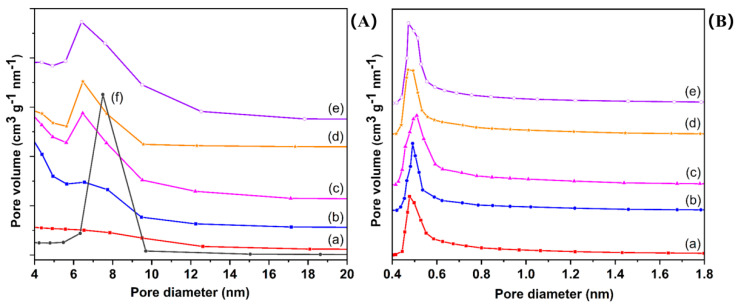
Mesopore size distributions (**A**) obtained by BJH method for (a) UiO-66-Br_2_, (b) U6B2S1, (c) U6B2S3, (d) U6B2S5, (e) U6B2S7, and (f) SBA-15, as well as their respective micropore size distributions (**B**) obtained by the HK method.

To further investigate the impact of SBA-15 on the growth of UiO-66-Br_2_, the mesopore and micropore size distributions of the samples were graphically represented in Figure 3. The BET surface areas, total pore volumes, and micropore volumes of the samples were presented in Table 1. The hybrid material exhibits a reduced total pore volume when compared to SBA-15 (0.77 cm^3^/g), yet it is marginally higher than pristine UiO-66-Br_2_. Additionally, the most probable pore diameters of the mesopores in the hybrid material are smaller than that of SBA-15, suggesting partial obstruction of the mesopores by UiO66-Br_2_ crystal fillings. These observations suggest that the formation of the MOF took place on both the internal and external surfaces of the SBA-15 material. Moreover, the total pore volumes of the composites exhibited a positive correlation with the quantity of SBA-15, suggesting that elevated concentrations of SBA-15 could potentially result in underutilization of the volumes. The integration of UiO66-Br_2_ with SBA-15 led to an increase in micropore volumes in the hybrid materials, indicating a synergistic interaction between the two materials. Therefore, it was hypothesized that SBA-15 served as a template for the growth of MOF crystals, with the formation of new micropores attributed to interactions between surface silanol groups and metal centers. In comparison to other composite materials, U6B2S5 demonstrates better structural attributes, evidenced by a micropore volume of 0.26 cm^3^/g and a high surface area value of 607.2 m^2^/g. The adsorption capacity of the materials is influenced by the BET surface area and pore size distribution, which also determine the samples’ capacity for selective CH_4_ adsorption.

### 2.3. SEM and TEM Images

**Figure 4:**
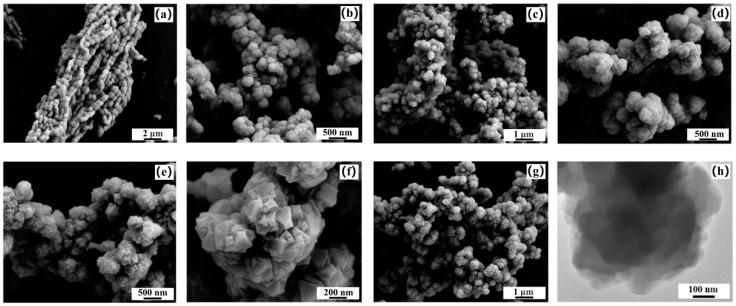
SEM images of (**a**) SBA-15, (**b**) UiO-66-Br_2_, (**c**) U6B2S1, (**d**) U6B2S3, (**e**,**f**) U6B2S5, and (**g**) U6B2S7 and TEM images of (**h**) U6B2S5.

SEM and TEM images were applied to study the morphologies of UiO-66-Br_2_, U6B2S1, U6B2S3, U6B2S5, U6B2S7, and SBA-15, which are presented in Figure 4. As shown in Figure 4a, the SBA-15 particles exhibited a rod-like morphology, with dimensions ranging from 0.5 to 1 μm in width and 1 to 2 μm in length, respectively. The as-synthesized UiO-66-Br_2_ exhibited an aggregated cauliflower-like morphology as opposed to the typical crystalline structure. However, it was observed that the composites exhibited a distinct morphology compared to both individual components, suggesting that the inclusion of SBA-15 rods had a significant impact on the structural characteristics of the resulting composites. As the amount of SBA increases, the crystal grains shapes observed in hybrid materials become more and more regular. For U6B2S5 (Figure 4e,f), most crystal grains show a very regular morphology. Based on the aforementioned analysis, it was determined that modifying the concentration of SBA-15 in the composites could lead to changes in morphology and a decrease in crystal particle size. The coordination between the -OH groups of SBA-15 and the Zr(Ⅳ) metal ions of UiO-66-Br_2_ facilitated the controlled crystal growth of the MOF, with subsequent platelet attachment to the silica surface. Additionally, the presence of SBA-15 imposed restrictions on the structural flexibility during the expansion of the UiO-66-Br_2_ framework, leading to the formation of smaller crystals. The TEM images presented in Figure 4h provide a clearer understanding of the phase distributions of UiO-66-Br_2_ crystals within the SBA-15 matrix. The U6B2S5 composite displays a stratified microstructure, indicating the organized placement of MOF platelets on the SBA-15 surface. Furthermore, the porous nature of SBA-15 remains intact throughout the composite formation process. These observations are consistent with the results obtained from PXRD and N_2_ adsorption–desorption measurements.

### 2.4. FT-IR Spectroscopy

**Figure 5:**
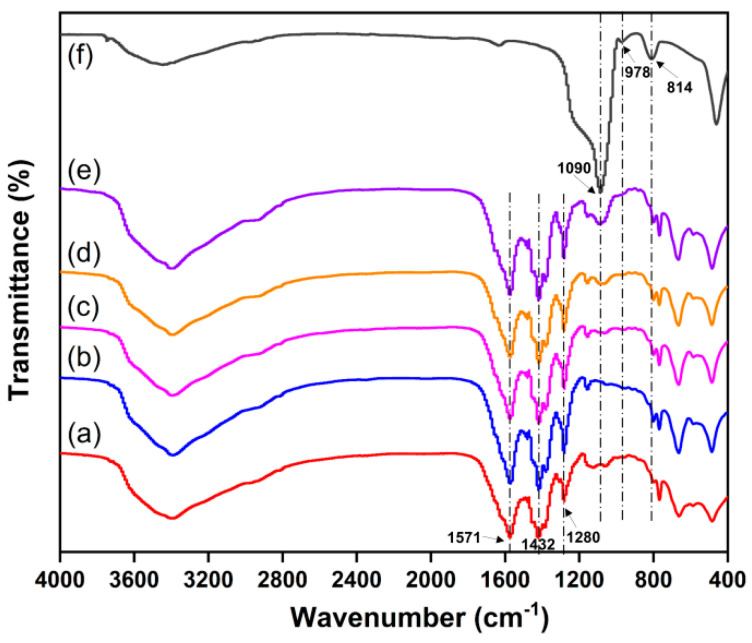
FT-IR spectra of (**a**) UiO66-Br_2_, (**b**) U6B2S1, (**c**) U6B2S3, (**d**) U6B2S5, (**e**) U6B2S7, and (**f**) SBA-15.

The FT-IR spectra of all samples are shown in Figure 5. In the case of UiO-66-Br_2_ (Figure 5a), the characteristic absorption at 1571 and 1280 cm^−1^ can be assigned to the asymmetric and symmetric stretching vibrations of carboxyl groups present in the H_2_BDC-Br_2_ ligands [45]. The absorption at 1432 cm^−1^ should be attributed to the C=C skeletal ring vibration of aromatic groups. For SBA-15 (Figure 5f), the strong peak at 1090 cm^−1^ is associated with the asymmetric stretching of the Si-O-Si bonds in the silicon–oxygen tetrahedral structure, while a weaker band at 804 cm^−1^ corresponds to the symmetric stretching vibrations. The distinctive vibrational band attributed to Si-OH groups was observed at around 978 cm^−1^ [46]. Analysis of the FT-IR spectra depicted in Figure 5b–e revealed a similarity between the spectra of all UiO-66-Br_2_/SBA-15 composites and that of pure UiO-66-Br_2_. Despite the relatively low intensity of the two adsorption bands corresponding to Si-O-Si, their presence in the composites was confirmed as evidenced by their detectability.

### 2.5. Thermogravimetric Analysis

**Figure 6:**
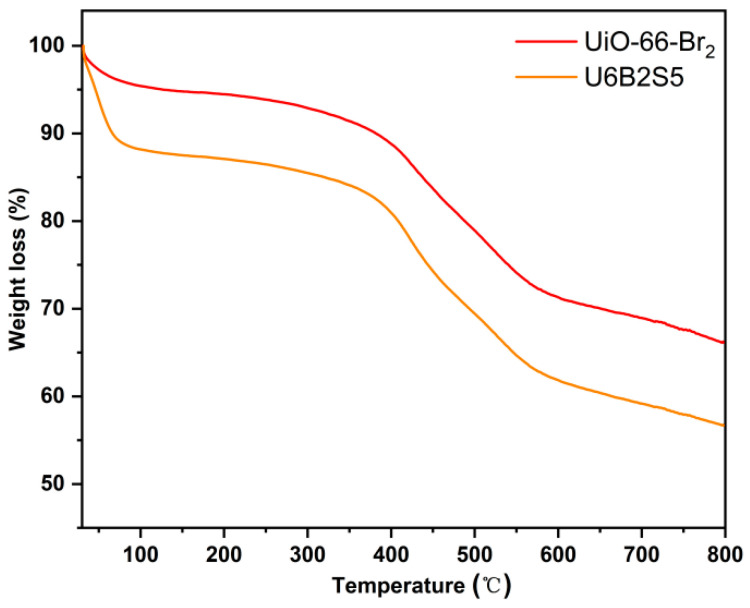
TG curves of UiO66-Br2 and U6B2S5 under N_2_ condition (ramping rate: 20 °C/min^−1^).

Thermal stability of materials is one of the key factors that determine their suitability for practical CH_4_/N_2_ separation applications. Therefore, thermogravimetric measurements for UiO-66-Br_2_ and U6B2S5 were performed in the temperature range from ambient temperature to 800 °C. As shown in Figure 6, it can be found that the two samples exhibited similar trend of thermogravimetric curves with the initial weight loss stage, attributed to the removal of adsorbed solvent and water molecules, occurring at temperatures below 100 °C. Subsequent weight loss was attributed to the decomposition of H_2_BDC-Br_2_ ligands and the collapse of the UiO-66-Br_2_ structure.

### 2.6. Adsorption and Separation Performance

**Figure 7:**
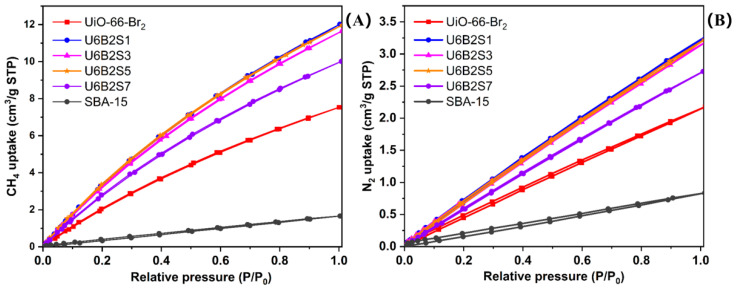
Adsorption and desorption equilibrium isotherms of CH4 (**A**) and N_2_ (**B**) for the samples.

In order to analyze the adsorption and separation performance, experimental measurements were conducted on the single-component adsorption isotherms of CH_4_ and N_2_ at 298 K with pressures up to 1.0 bar, as depicted in Figure 7. Throughout the entire pressure range examined, the CH_4_ loading was significantly higher than that of N_2_, with the latter gas displaying a nearly linear increase.

**Figure 8:**
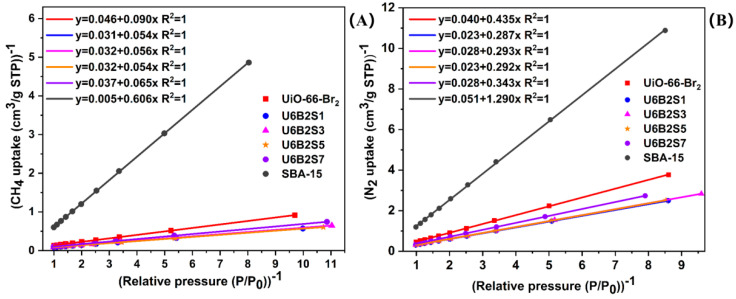
(1/V) versus (1/P) plots of CH_4_ (**A**) and N_2_ (**B**) for the samples with symbols denoting experimental values and lines representing linear fitting to the Langmuir equation.

The adsorption capacities of CH_4_ and N_2_ for the samples under vacuum swing adsorption (VSA) conditions, specifically within the low-pressure range of 0.1–1 bar, are detailed in Table 2. This pressure range is representative of the typical conditions utilized for coalbed methane (CBM) enrichment. In this condition, the Langmuir model (Equation (1)) is employed to analyze the isotherms and establish the relationship between CH_4_ and N_2_ adsorption [47]. Equation (2) can be obtained through the transformation of Equation (1) into its reciprocal form, thereby illustrating the linear correlation between the 1/V and 1/P. Using Equation (2), the adsorption values in Table 2 were processed and plotted as shown in Figure 8.

(1) $$V=\frac{V_{m}\cdot B\cdot P}{1+B\cdot P}$$

(2) $$\frac{1}{V}=\frac{1}{B\cdot V_{m}}\cdot \frac{1}{P}+\frac{1}{V_{m}}$$

Understanding the absorption equilibrium selectivity and adsorption capacity coefficient of an adsorbent are the major factors affecting its adsorption selectivity efficiency. By utilizing the fitting equations depicted in Figure 8, the slope b of each fitting equation can be ascertained. By applying Equation (3), the adsorption equilibrium selectivity of CH_4_/N_2_ (*α*_CH4/N2_) can be computed. X_CH4_ or X_N2_ and γ_CH4_ or γ_N2_ denote the molar fractions of CH4 or N2 in the adsorbed and gas phases, respectively. The adsorption capacity coefficient (*W*_CH4/N2_) can be calculated from the data in Table 3 using the Equation (4).

(3) $$\alpha_{CH_{4}/N_{2}}=\frac{X_{CH_{4}}}{X_{N_{2}}}\cdot \frac{\gamma_{N_{2}}}{\gamma_{{CH}_{4}}}=\frac{b_{N_{2}}}{b_{{CH}_{4}}}$$

(4) $$W_{CH_{4}/N_{2}}=\frac{{∆}_{CH_{4}}}{{∆}_{N_{2}}}=\frac{V_{{{CH}_{4}}^{\left(Adsorption\;1.0\;bar\right)}}-V_{{{CH}_{4}}^{\left(Adsorption\;0.1\;bar\right)}}}{V_{{N_{2}}^{\left(Adsorption\;1.0\;bar\right)}}-V_{{N_{2}}^{\left(Adsorption\;0.1\;bar\right)}}}$$

It is important to acknowledge that the parameter *S* for adsorbent selection holds greater utility in the evaluation and selection of adsorbents and can subsequently be calculated using Equation (5). The parameter *S* for adsorbent selection serves as a useful tool for evaluating and comparing the adsorption efficiency of different absorbents. As the adsorption performance of the adsorbent improves, the value of *S* also increases.

(5) $$S_{CH_{4}/N_{2}}=\alpha_{CH_{4}/N_{2}}\cdot \;W_{CH_{4}/N_{2}}$$

According to the data presented in Table 4, the samples can be arranged in descending order based on the adsorption selection parameter *S*_CH4/N2_ as follows: U6B2S5 > U6B2S7 > U6B2S3 > U6B2S1 > UiO-66-Br_2_ > SBA-15. The hybrid materials of UiO-66-Br_2_/SBA-15 demonstrate enhanced adsorption selectivity compared to the pristine UiO-66-Br_2_, suggesting that the combination of MOF structure and SBA-15 enhances the selectivity for CH_4_ adsorption. Among the samples, U6B2S5 demonstrates the highest adsorption selectivity, as indicated by its *α*_CH4/N2_ value of 5.45 and *S*_CH4/N2_ value of 20.06. Interestingly, in the context of equilibrium adsorption, the *S*_CH4/N2_ value of U6B2S5 surpasses the values reported for MOF-1/SBA-15, MOF-2/SBA-15, 5A Zeolite, MOF-5, and MOF-177 under the same conditions [48,49]. These observations demonstrate that U6B2S5 is a robust material with excellent performance for CH_4_/N_2_ separation.

## 3. Materials and Methods

### 3.1. Materials

Zirconium chloride (ZrCl_4_), 2,5-dibromoterephthalic acid (H_2_BDC-Br_2_), Pluronic P123 triblock copolymer (EO_20_PO_70_EO_20_, average Mw = 5800), glycerol, and tetraethyl orthosilicate (TEOS) were procured from Sigma-Aldrich and utilized in their original form unless specified otherwise. Additional solvents were commercially accessible, and all reagents met the standards of analytical grade.

### 3.2. Synthesis of SBA-15

SBA-15 was synthesized following a previously reported procedure with slight modifications [50]. Specifically, 4.0 g of P123 was dissolved in 120 mL of hydrochloric acid aqueous solution (2.4 mol/L) and stirred for 4 h at 40 °C until a clear and homogeneous solution was obtained. Subsequently, 8.5 g of TEOS was incrementally added to the reaction mixture under stirring conditions, followed by incubation at 40 °C for a duration of 24 h. In the following step, the temperature was raised to 80 °C, and the mixture was aged under static conditions for 48 h. The white precipitates were subjected to filtration, washed extensively with deionized water, and subsequently dried at a temperature of 60 °C for a duration of 12 h. Finally, the P123 template was eliminated through calcination of the SBA-15 precursor at a temperature of 550 °C for a duration of 6 h.

### 3.3. Synthesis of UiO-66-Br_2_

Pure UiO-66-Br_2_ was synthesized following established procedures outlined in the literature [44]. Generally, ZrCl_4_ (0.93 g, 4 mmol), 2,5-dibromoterephthalic acid (H_2_BDC-Br_2_, 1.93 g, 8 mmol), and concentrated HCl (0.67 mL) are dissolved in N, N’-dimethylformamide (DMF) (24 mL). The resulting solution are then subjected to heating in a 100 mL autoclave at 220 °C for a duration of 16 h. Upon cooling of the reaction system, the product is isolated. The resulting substance is subjected to multiple washings with dimethylformamide (DMF) and ethanol, followed by vacuum drying at 60 °C for a specific duration, resulting in the successful acquisition of the desired final product.

### 3.4. Synthesis of UiO-66-Br_2_/SBA-15

The UiO-66-Br_2_/SBA-15 composites were synthesized using a solvothermal approach. Typically, a specified quantity of SBA-15 would be dispersed in a solution of zirconium (IV) chloride in DMF, followed by sonication for a duration of 30 min. Subsequently, a well-dissolved DMF solution of H_2_BDC-Br_2_ is introduced, along with a specified quantity of concentrated HCl. The resulting suspension is vigorously stirred for an additional 30 min prior to transfer into an autoclave. The synthesis of UiO-66-Br_2_/SBA-15 composites adhered to the procedures outlined for UiO-66-Br_2_. Specifically, different quantities of SBA-15 are included in the composite synthesis process, with concentrations ranging from 1, 3, 5 to 7 weight percent relative to the mass of ZrCl_4_. The resulting hybrid materials were denoted as U6B2S1, U6B2S3, U6B2S5, and U6B2S7, respectively.

### 3.5. Characterization

The samples’ phase composition was assessed using X-ray powder diffraction (PXRD: ultimaIV, Rigaku, Tokyo, Japan) at a tube voltage of 40 kV and tube current of 40 mA, utilizing Cu Kα radiation (1.5418 Å). Scans were conducted within the small-angle range of 0.5–5.0° and the wide-angle range of 5–40°. The specific surface area and pore volume of each sample were determined by N_2_ adsorption measurements (BSD-660M A6M, BSD instrument, Beijing, China) at 77 K following pretreatment of the samples at 493 K under vacuum. The microstructure of the sample was examined using scanning electron microscopy (SEM: Zeiss Gemini sigma 300, Oberkochen, Germany) at a working voltage of 5.0 kV. The transmission electron microscopy (TEM: FEI Talos F200S, Hillsboro, OR, USA) analysis was conducted at an operating voltage of 200 kV. Fourier transform infrared (FT-IR) spectroscopy of the samples was obtained by KBr tableting method and a Thermo Nicolet iS5 spectrophotometer (Waltham, MA, USA). Thermogravimetric analysis was conducted utilizing a NETZSCH TG 209F3 instrument (TGA: NETZSCH, Bavaria, Germany), with temperature varying from ambient temperature to 800 °C at a heating rate of 10 °C/min in the presence of a dynamic N_2_ atmosphere.

### 3.6. Adsorption Performance

A room temperature and atmospheric pressure adsorption and desorption experiment was conducted on the BSD-660 instrument, with a pressure range of 0–1 bar. An amount of 450 mg of sample was taken and placed in a sample tube. After heating to 200 °C and vacuum pretreatment for 3 h, the pressure change was detected by a pressure sensor to calculate the adsorption and desorption amount. High purity He (≥99.999%), CH_4_ (≥99.999%), and N_2_ (≥99.9999%) were used to adsorb gases.

## 4. Conclusions

In this study, composite materials consisting of rod-shaped SBA-15-based UiO-66-Br_2_ have been synthesized and characterized. The structural characterization results revealed that MOF nanocrystals grew on the surfaces of the SBA-15 matrix, leading to the formation of composites and the emergence of new micropores through interactions between surface silanol groups. The adsorption performance test indicated that the U6B2S5 sample exhibited superior CH_4_/N_2_ adsorption selectivity, as evidenced by an adsorbent selection parameter (*S*) of 20.06, suggesting promising prospects for practical utilization. In summary, this study not only presents a potential candidate for CH_4_/N_2_ separation but also offers valuable insights for the development of top-performing MOF/SBA-15 composites.

## Figures and Tables

**Table 1:** Pore structure parameters of the samples determined from the nitrogen adsorption–desorption results.

Samples	BET Surface Area (m^2^/g)	Total Pore Volume (cm^3^/g)	Micropore Volume (cm^3^/g)
UiO-66-Br_2_	515.9	0.28	0.21
U6B2S1	626.2	0.29	0.23
U6B2S3	610.2	0.31	0.24
U6B2S5	607.2	0.32	0.26
U6B2S7	521.7	0.34	0.16
SBA-15	550.2	0.77	-

**Table 2:** The low-pressure (0.1–1 bar) adsorption capacity of CH_4_ and N_2_ for the samples.

	CH_4_ Adsorption		N_2_ Adsorption		Sample	CH_4_ Adsorption		N_2_ Adsorption	
	P/P_0_	V_CH4_ (cm^3^/g)	P/P_0_	V_N2_ (cm^3^/g)		P/P_0_	V_CH4_ (cm^3^/g)	P/P_0_	V_N2_ (cm^3^/g)
UiO-66-Br_2_	0.10	1.09	0.12	0.27	U6B2S5	0.09	1.65	0.12	0.40
	0.19	1.93	0.20	0.45		0.19	3.12	0.20	0.66
	0.30	2.85	0.30	0.66		0.29	4.59	0.30	0.99
	0.40	3.66	0.40	0.88		0.40	6.03	0.39	1.31
	0.50	4.41	0.50	1.09		0.50	7.12	0.49	1.62
	0.60	5.09	0.60	1.31		0.60	8.23	0.59	1.93
	0.70	5.76	0.70	1.51		0.69	9.13	0.69	2.25
	0.80	6.36	0.80	1.72		0.82	10.35	0.81	2.60
	0.90	6.94	0.90	1.93		0.90	11.05	0.90	2.86
	1.00	7.53	1.02	2.18		1.01	11.97	1.01	3.22
U6B2S1	0.10	1.75	0.12	0.40	U6B2S7	0.09	1.35	0.13	0.37
	0.18	3.08	0.20	0.67		0.19	2.59	0.20	0.59
	0.30	4.74	0.29	1.00		0.31	4.02	0.29	0.83
	0.40	5.93	0.40	1.34		0.40	4.99	0.40	1.14
	0.50	7.17	0.50	1.67		0.50	5.91	0.49	1.39
	0.59	8.14	0.60	1.99		0.59	6.80	0.59	1.65
	0.71	9.31	0.70	2.30		0.70	7.69	0.69	1.92
	0.80	10.16	0.80	2.60		0.80	8.49	0.79	2.18
	0.90	11.13	0.90	2.91		0.90	9.21	0.89	2.44
	1.00	12.01	1.02	3.28		1.01	10.01	1.01	2.73
U6B2S3	0.09	1.54	0.10	0.35	SBA-15	0.12	0.21	0.12	0.09
	0.19	2.99	0.19	0.65		0.20	0.33	0.20	0.15
	0.29	4.48	0.29	0.98		0.30	0.49	0.29	0.23
	0.42	5.98	0.39	1.29		0.39	0.65	0.39	0.31
	0.50	6.92	0.49	1.61		0.50	0.83	0.49	0.39
	0.60	7.99	0.60	1.94		0.60	0.99	0.59	0.47
	0.70	8.96	0.70	2.24		0.70	1.15	0.69	0.56
	0.80	9.88	0.80	2.54		0.80	1.31	0.79	0.64
	0.90	10.72	0.90	2.83		0.90	1.49	0.89	0.73
	1.01	11.64	1.02	3.20		1.00	1.66	1.01	0.84

**Table 3:** Summary of the pure component adsorption behaviors of the samples.

Sample	Adsorption Equilibrium Amount (cm^3^/g)			
	Adsorption Pressure of 1.0 bar		Desorption Pressure of 0.1 bar	
	V_CH4_ (cm^3^/g)	V_N2_ (cm^3^/g)	V_CH4_ (cm^3^/g)	V_N2_ (cm^3^/g)
UiO-66-Br_2_	7.53	2.18	1.33	0.30
U6B2S1	12.01	3.28	2.14	0.44
U6B2S3	11.64	3.20	1.71	0.39
U6B2S5	11.97	3.22	1.53	0.39
U6B2S7	10.01	2.73	1.46	0.34
SBA-15	1.66	0.84	0.25	0.14

**Table 4:** Summary of parameters related to CH_4_/N_2_ adsorption selectivity of samples.

Sample	Parameters Related to the Adsorption Selectivity			
	*α*_CH4/N2_	*W*_CH4/N2_	*S*_CH4/N2_	Reference
SBA-15	2.13	2.02	4.30	this work
UiO-66-Br2	4.84	3.31	16.02	this work
U6B2S1	5.30	3.46	18.34	this work
U6B2S3	5.24	3.54	18.55	this work
U6B2S5	5.45	3.68	20.06	this work
U6B2S7	5.27	3.58	18.86	this work
5A Zeolite	0.94	-	0.81	[49]
MOF-5	1.13		0.67	[49]
MOF-177	4.00		8.45	[49]
MOF-1/SBA-15	2.17	2.19	4.75	[50]
MOF-2/SBA-15	3.44	3.24	11.1	[50]

## Data Availability

Data is contained within the article, further inquiries can be directed to the corresponding author.
